# TAp63γ influences mouse cartilage development

**DOI:** 10.18632/aging.103190

**Published:** 2020-05-11

**Authors:** Qian Wang, Na Li, Fangzhou Chen, Ruoxuan Hei, Junxia Gu, Yaojuan Lu, Lichun Sun, Qiping Zheng

**Affiliations:** 1 Department of Hematological Laboratory Science, Jiangsu Key Laboratory of Medical Science and Laboratory Medicine, School of Medicine, Jiangsu University, Zhenjiang 212013, China; 2 Department of Blood Transfusion, The First Affiliated Hospital of Anhui Medical University, Hefei 230022, China; 3 Shenzhen Academy of Peptide Targeting Technology at Pingshan, and Shenzhen Tyercan Bio-pharm Co., Ltd., Shenzhen 518118, China; 4 Department of Medicine, School of Medicine, Tulane Health Sciences Center, New Orleans, LA 70112, USA

**Keywords:** TAp63γ, ATDC5, chondrocyte hypertrophy, endochondral ossification, transgenic studies

## Abstract

Depletion of tumor protein p63 results in severe epithelial as well as limb defects in mice, suggesting that p63 is also required for endochondral ossification during long bone development. A key stage in endochondral ossification is chondrocyte hypertrophy, which has been associated with elevated levels of the p63 variant TAp63γ. To investigate the role of TAp63γ in chondrocyte differentiation and maturation, we developed stable TAp63γ expressing ATDC5 cells. Compared to control cells, *TAp63γ* cells showed significant upregulation of Col10a1 after 4 and 7 days in culture. Moreover, alkaline phosphatase, Alizarin red, and Alcian blue staining were stronger in *TAp63γ* cells, suggesting that TAp63γ promotes chondrocyte proliferation, hypertrophic differentiation, and possibly matrix mineralization. To investigate the *in vivo* function of TAp63γ during endochondral bone formation, we established transgenic mice that express flag-tagged *TAp63γ* driven by *Col10a1* regulatory elements. Skeletal staining of transgenic mice at postnatal day 1 showed accelerated ossification in long bone, tail, and digit bones compared to wild-type littermates. Furthermore, Sox9 expression was reduced and Runx2 expression was increased in the proliferative and/or hypertrophic zones of these mice. Altogether, these results suggest that TAp63γ promotes endochondral ossification and skeletal development, at least partially via controlling chondrocyte differentiation and maturation.

## INTRODUCTION

Tumor protein p63 is a member of the p53 tumor suppressor family and plays a role in ectoderm differentiation and in the basal regenerative layers of epithelial tissues in the adult [1]. p63 is also important for formation of the limb bud which is developed from the mesoderm. Knockout of p63 in mice results in defects in limb, craniofacial, and epithelial development. Specific defects include absent or truncated limbs and lack of epithelial tissues and their derivatives, including mammary, lachrymal, and salivary glands, due to loss of ectodermal stem cells [2–4]. In addition to limb loss and truncation, depletion of p63 in mice results in obvious developmental abnormalities, which include severe skin, heart, and germ line defects [5, 6].

Heterozygous missense mutations of the *P63* gene in humans are closely related to ectrodactyly, ectodermal dysplasia, and cleft lip/palate (EEC) syndrome [7, 8]. Moreover, pathogenic *P63* mutations are associated with four syndromes: ankyloblepharon-ectodermal defects-cleft lip/palate syndrome (AEC), acro-dermato-ungual-lacrimal-tooth syndrome (ADULT), limb mammary syndrome (LMS), and Rapp-Hodgkin syndrome (RHS) [9]. Novel *P63* mutations in humans have also been associated with split-hand/foot malformation (SHFM) and hypodontia [10–12]. In adulthood, cartilage degeneration is known to cause osteoarthritis (OA), which is the most common joint disorder. Interestingly, age-related OA development is suppressed in p63 conditional knockout mice [13]. Moreover, upregulation of p63 in the cartilage tissues of OA patients inhibits chondrocyte autophagy and contributes to OA progression [14], suggesting a role of p63 during articular cartilage degeneration.

As a member of the p53 tumor suppressor family, altered p63 expression is related to tumor occurrence. *P63* mutations result in cell proliferation and apoptosis inhibition in a variety of tumor cell types, such as giant cell tumors of the bone, chondrosarcoma malignancies, and squamous cell carcinoma [15–17]. Notably, p63 heterozygous mice have a shortened life-span, suggesting that loss of p63 induces cellular senescence and causes features of accelerated aging. p63 deficiency also stimulates cellular senescence, evidenced by enhanced expression of the senescence markers SA-beta-gal, PML, and p16^INK4a^ [18]. Interestingly, TAp63 isoforms in response to genotoxic stress have been demonstrated. Both in primary cells and tumor cell lines, TAp63 isoforms can regulate the expression of GLS2 which is important in the cellular antioxidant pathway [19].

There are many p63 variants, including TAp63-α/β/γ and ΔNP63-α/β/γ, which encode various p63 isoforms with intact or truncated N- and/or C-terminal domains. Of these variants, TAp63α and TAp63γ are highly expressed in mouse articular chondrocytes and primary costal chondrocytes [13]. Our previous studies demonstrated that TAp63α plays a modest role in endochondral ossification through genes (such as *Alp*, *Ank*, *Bcl2*, *Sox9*, etc.) relevant to matrix mineralization, chondrocyte maturation, and apoptosis. In contrast, phenotypic analyses of *Col2a1-ΔNp63α/ TAp63α* and *Col10a1-ΔNp63α/ TAp63α* transgenic mice suggest an insignificant role of ΔNp63α during embryonic skeletal development. These results suggest that other p63 isoforms may play more vital roles in skeletal formation [20, 21].

Notably, we have recently detected increase of the γ variant, TAp63γ, during chondrocyte hypertrophy. Chondrocyte hypertrophy is a critical stage of endochondral ossification that has been implicated as a primary driver of long bone growth [22, 23]. We therefore hypothesized that TAp63γ may play an essential role in long bone development targeting chondrocyte hypertrophy. This study investigated both the *in vitro* and *in vivo* effects of TAp63γ on chondrocyte proliferation, differentiation, and maturation, so as to provide an experimental and theoretical basis for further studies geared toward understanding the mechanism of bone and cartilage development and aging-related degenerative disease.

## RESULTS

### Col10a1-TAp63γ expression plasmid and establishment of TAp63γ expressing ATDC5 stable cell lines.

**Figure 1:**
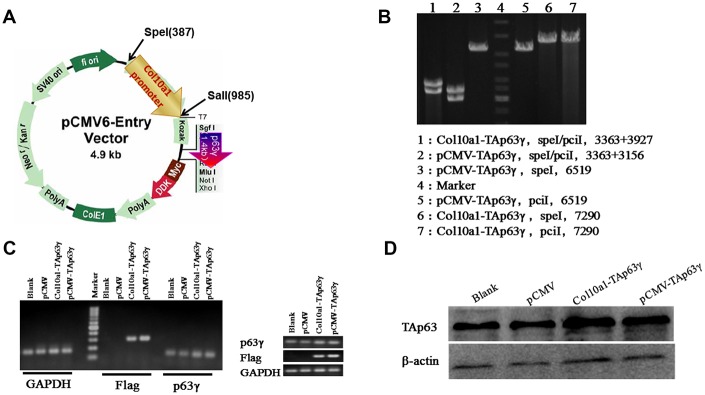
***Col10a1-TAp63γ* expression plasmid and establishment of stable *TAp63γ* expressing ATDC5 cell lines.** (**A**) pCMV-*TAp63γ* and its derivative *pCol10a1-TAp63γ* expression plasmids are shown. Enzyme restriction sites for cloning are also shown. (**B**) Enzyme digestion confirmed the integration of *TAp63γ* in designated stable cell lines. (**C**) PCR using *p63* and Taq sequence-specific primers confirmed the integration of *TAp63γ* into the stable cell lines: pCMV-*TAp63γ* and *Col10a1-TAp63γ*. (**D**) Western blot results further confirmed expression of TAp63γ in designated stable lines.

The *TAp63γ* expression plasmid (pCMV-*TAp63γ*) MR227536 was purchased from Origene. *TAp63γ* was cloned into the pCMV-entry between multiple restriction sites: *SgfI* and *MluI*. *Col10a1-TAp63γ* was generated by replacing the CMV promoter with the hypertrophic chondrocyte-specific *Col10a1* enhancer/promoter element [15] via *SpeI* and *SalI* double digestion (Figure 1A). Figure 1B shows confirmation of the clones by enzyme digestion.

Stable lines expressing either pCMV-*TAp63γ*, *Col10a1-TAp63γ*, or the pCMV-entry vector control were generated by transient transfection followed by G418 selection. Colonies expressing *TAp63γ* were identified by PCR using *p63* and Taq sequence-specific primers (Figure 1C). The primers used for RT-PCR are listed in Table 1. Western blot was used to confirm TAp63γ expression (Figure 1D). Together, the PCR and western blot results demonstrate successful generation of stable *TAp63γ* (pCMV-*TAp63γ* and *Col10a1-TAp63γ*) ATDC5 cell lines.

**Table 1:** Primers designed for PCR.

**Name**	**RefseqID**	**Sense primer (5’-3’)**	**Antisense primer (5’-3’)**	**Amplicon (bp)**
*Gapdh*	NM_008084	ACCCAGAAGACTGTGGATGG	CACATTGGGGGTAGGAACAC	171
*Col10a1*	NM_009925	GCAGCATTACGACCCAAGATC	TCTGTGAGCTCCATGATTGC	201
*p63γ*	NM_001127259	GTATCGGACAGCGCAAAGAACG	CTGGTAGGTACAGCAGCTCATC	123
p63γ*-*flag		ACCAGTGAGGTCGTGAGA	TCATTTGCTGCCAGATCCTCTT	311

### TAp63γ upregulates Col10a1 expression in ATDC5 cells

**Figure 2:**
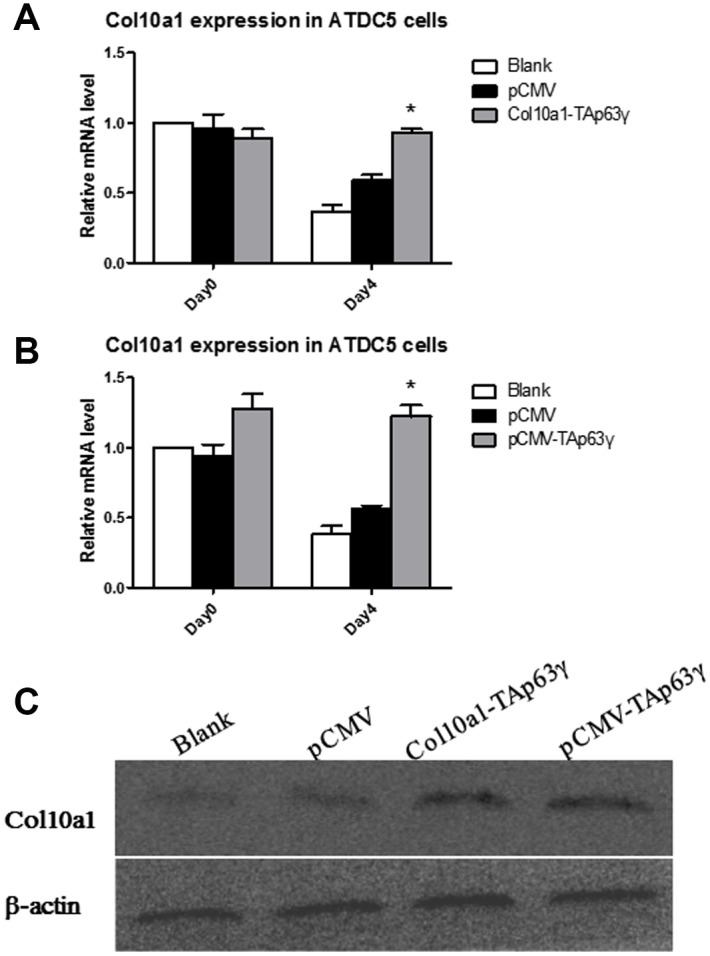
**TAp63γ upregulates Col10a1 expression in ATDC5 cells.** (**A**) ATD5C cells were harvested for RNA isolation on day zero or after 4 days in culture. *Col10a1* showed significant elevation in *Col10a1-TAp63γ* stable cell lines compared with the controls after 4 days in culture. (**B**) ATD5C cells were harvested for RNA isolation on day zero or after 4 days in culture. *Col10a1* showed significant elevation in pCMV-*TAp63γ* stable cell lines compared with the controls after 4 days in culture. (**C**) The protein levels of Col10a1 in *TAp63γ* stable cell lines also showed upregulation of Col10a1 compared to blank and pCMV controls by western blot analysis.

ATDC5 cells stably integrated with blank, vector, or *TAp63γ* plasmids were subjected to prolonged culture, and RNA was extracted for expression analysis. cDNA was synthesized, and expression of *Col10a1* and *Gapdh* was evaluated. After 4 days in culture, *Col10a1* was significantly elevated in both *Col10a1-TAp63γ* and pCMV-*TAp63γ* stable cell lines compared with the controls (Figure 2A, 2B). Protein levels of Col10a1 were also elevated in the *Col10a1-TAp63γ* and pCMV-*TAp63γ* stable cell lines (Figure 2C). Similar results were observed in cells cultured for 7 days (data not shown).

### In vitro effect of TAp63γ on chondrocyte proliferation

**Figure 3:**
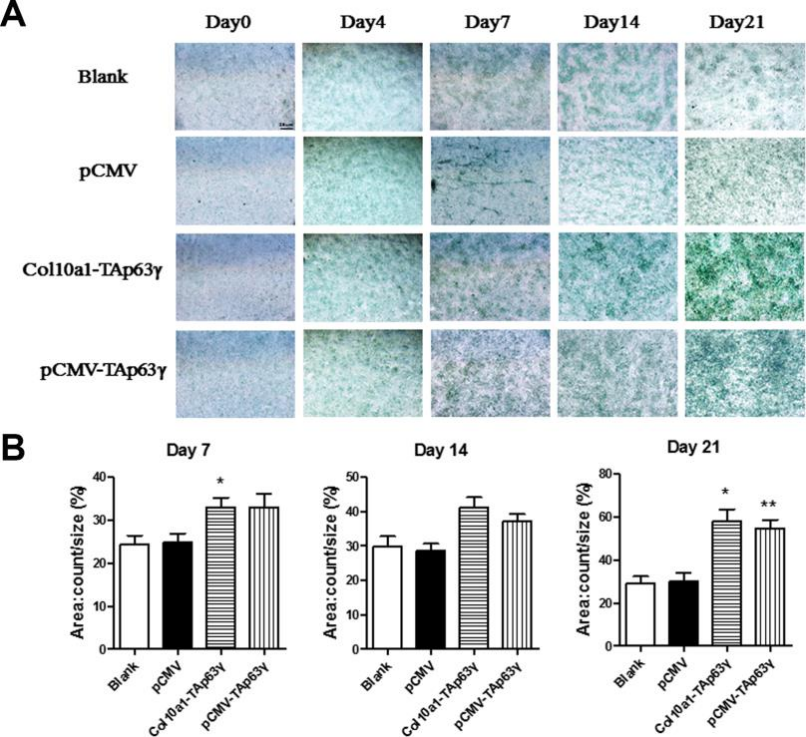
***In vitro* effect of TAp63γ on chondrocyte proliferation.** (**A**) ATD5C cells were cultured for 0, 4, 7, 14, or 21 days and stained with Alcian blue. After 7 days in culture, the staining intensity of *TAp63γ* stable cell lines was much stronger than the blank and vector controls. Scale bar, 25 μm. (**B**) Sum object area of the staining by densitometry analysis (n=3, * p<0.05, ** p<0.01).

Cells cultured for 0, 4, 7, 14, and 21 days were subjected to Alcian blue staining. Enhanced staining was observed in *TAp63γ* stable cell lines cultured for 7 days or longer compared to control cells (Figure 3), suggesting that TAp63γ promotes chondrocyte proliferation *in vitro*.

### TAP63γ potentially promotes hypertrophic differentiation of ATDC5 cells

**Figure 4:**
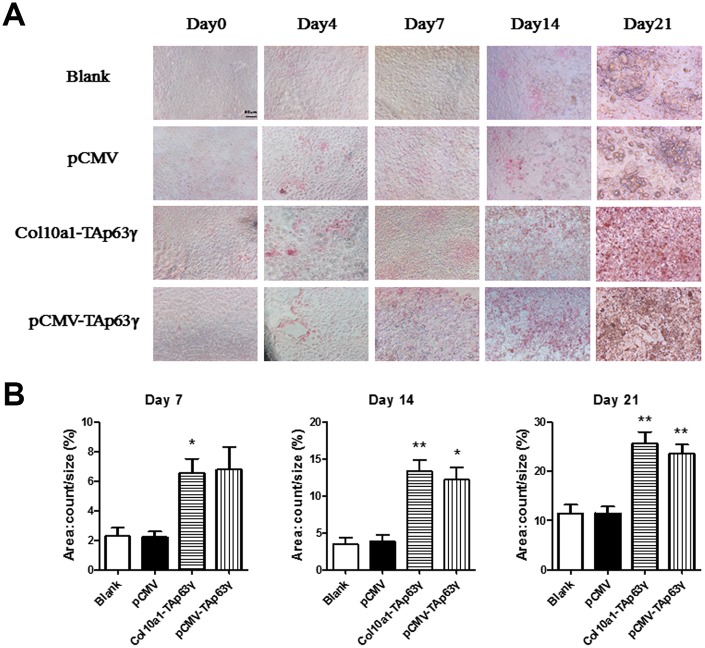
**TAp63γ promotes hypertrophic differentiation of ATDC5 cells.** (**A**) ATD5C cells were cultured for 0, 4, 7, 14, or 21 days and stained for ALP (alkaline phosphatase). After 7 days in culture, the staining intensity of *TAp63γ* stable cell lines was much stronger than the blank and vector controls. Scale bar, 50 μm. (**B**) Sum object area of the staining by densitometry analysis (n=3, * p<0.05, ** p<0.01).

Cells cultured for 0, 4, 7, 14, and 21 days were subjected to ALP (alkaline phosphatase) staining. Compared to control cells, no staining difference was initially observed in *TAp63γ* stable cell lines. However, after 7 days in culture, ALP staining intensity in *TAp63γ* stable cell lines was much stronger than the control cells (Figure 4), suggesting that TAp63γ may promote chondrocyte hypertrophic differentiation *in vitro*.

### In vitro effect of TAP63γ on matrix mineralization

**Figure 5:**
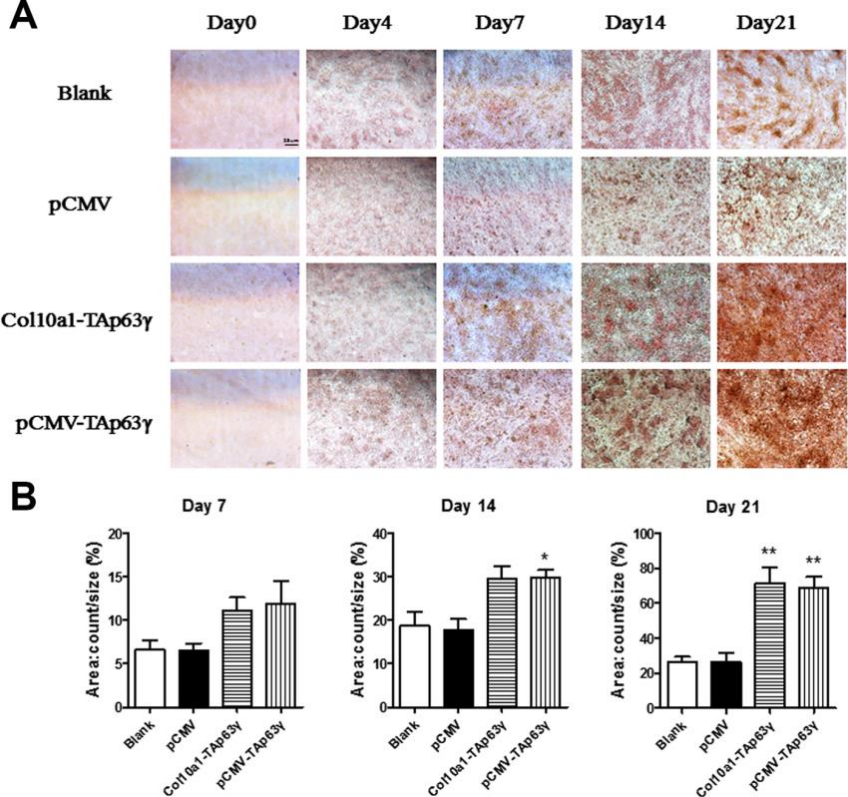
***In vitro* effect of TAp63γ on matrix mineralization.** (**A**) ATD5C cells were cultured for 0, 4, 7, 14, or 21 days and stained with Alizarin red. After 4 and 7 days in culture, enhanced Alizarin red staining was observed in both *Col10a1-TAp63γ* and pCMV-*TAp63γ* stable cell lines compared with the blank and vector controls. Scale bar, 25 μm. (**B**) Sum object area of the staining by densitometry analysis (n=3, * p<0.05, ** p<0.01).

We also performed Alizarin red staining in cells cultured for 0, 4, 7, 14, and 21 days. Enhanced staining was demonstrated in both *Col10a1-TAp63γ* and pCMV-*TAp63γ* stable cell lines compared with the control cells (Figure 5), suggesting that TAp63γ may promote matrix mineralization.

### Accelerated ossification in Col10a1-TAp63γ transgenic mice

**Figure 6:**
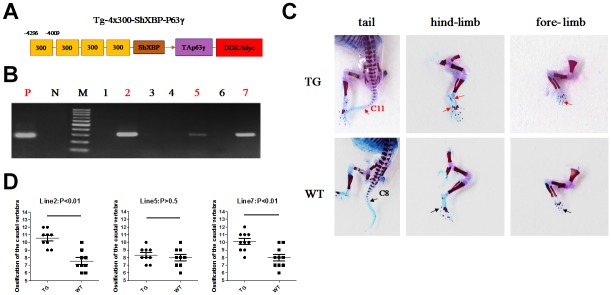
**Accelerated ossification in *Col10a1-TAp63γ* transgenic mice.** (**A**) *Col10a1* distal promoter and a shorter *Col10a1* basal promoter (ShXBP) (from −220 to +45 bp) were used to generate a p63-expressing transgenic construct. (**B**) PCR genotyping was performed for the *Col10a1-TAp63γ* transgenic mice using DNA prepared from skin. (**C**) For mice at postnatal day 1 (P1), ossification signals of the fore- and hind-limb digits were evaluated. Tail ossification signals were observed up to the 11^th^ caudal vertebra in transgenic mice and up to the 8^th^ caudal vertebra in WT mice. (**D**) The statistical analyses of the ossified caudal vertebrae from four *Col10a1-TAp63γ* transgenic mouse lines at P1 are presented. Line 2: n = 9; Line 5: n = 9; Line 7: n = 10.

The *Col10a1* distal promoter and the shorter *Col10a1* basal promoter (from −220 to +45 bp) elements were used to generate a p63γ expressing transgenic construct. Generation of transgenic mice was conducted at Lannuo Biotechnologies Wuxi, Inc (Figure 6A). PCR genotyping was performed for the *Col10a1-TAp63γ* transgenic mice using DNA prepared from skin. Lanes 2, 5, and 7 were *Col10a1-TAp63γ* transgenic mice, and lanes 1, 3, 4, and 6 were wild-type littermates (WT mice) (Figure 6B). Mice at postnatal day 1 (P1) were analyzed for ossification of the fore- and hind-limb digits. Ossification was apparent in some digits of transgenic mice but not in WT littermates. Moreover, tail ossification was evident up to the 11^th^ caudal vertebra in transgenic mice; whereas, significantly delayed ossification could only be observed up to the 8^th^ caudal vertebra in WT littermates in two of the transgenic mouse lines (p<0.05, Figure 6C, 6D).

### Histological analysis and immunofluorescent staining of Col10a1-TAp63γ transgenic mice

**Figure 7:**
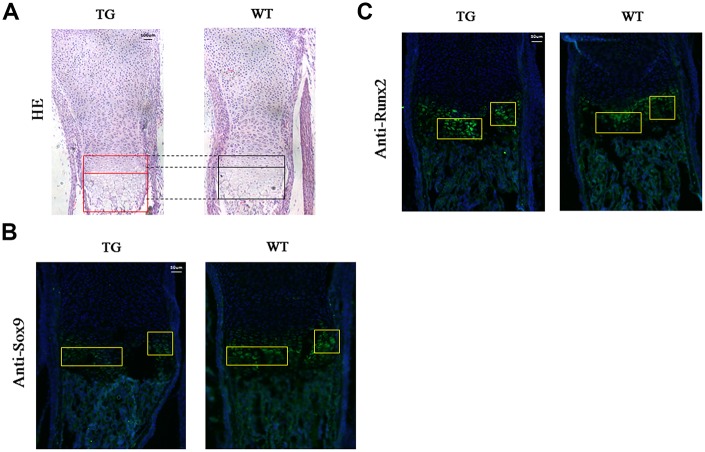
**Histological and immunofluorescent analysis of *Col10a1-TAp63γ* transgenic mice.** (**A**) The proliferative and hypertrophic zones of the limb cartilage in *Col10a1-TAp63γ* transgenic and WT mice were evaluated by hematoxylin and eosin (H&E) staining. Scale bar, 100 μm. (**B**) Sagittal sections of the distal humerus from both WT and transgenic mouse limbs at postnatal day 1 were subjected to immunofluorescent analysis using an anti-Sox9 antibody. Scale bar, 50 μm. (**C**) Sagittal sections of the distal humerus from both WT and transgenic mouse limbs at postnatal day 1 were subjected to immunofluorescent analysis using an anti-Runx2 antibody (label as Sox9 and some description of the findings). Scale bar, 50 μm.

Mouse limbs were collected at P1 stage and subjected to hematoxylin and eosin (H&E) or immunofluorescent staining. H&E staining demonstrated that the proliferative and hypertrophic zones of the limb cartilage were significantly increased in the transgenic mice compared to the WT littermates (Figure 7A). Sagittal sections of the distal humerus from both WT and transgenic mouse limbs at P1 were subjected to immunofluorescent analysis using an anti-Sox9 antibody. Reduced green fluorescence, indicating down-regulation of Sox9 expression, was observed in the proliferative and hypertrophic zones of transgenic mice compared to WT controls (Figure 7B). Meanwhile, increased green fluorescence, indicating up-regulation of Runx2 expression, was observed in the hypertrophic zone of transgenic mice compared to their littermate controls (Figure 7C).

## DISCUSSION

In addition to its epithelial effects, p63 is closely related to the occurrence and development of osteosarcoma, bone, cartilage, and osteoarthritis [4, 14–16, 20]. Although p63 has been implicated a role in bone development for nearly two decades, little effort has been given to increasing our understanding of the detailed mechanism of p63 action. There are six p63 isoforms that have been extensively studied and are known to play distinct roles during development and cancer formation [24, 25]. Generally, the transactivation (TA) domain isoforms (TAp63) act as transcription factors; whereas, the ΔNp63 isoforms act as dominant-negative inhibitors of TA isoforms. To date, how p63 and its variants function during specific stages of skeletal development remains unknown. We have previously shown moderate skeletal phenotypes in mice overexpressing TAp63α and ΔNp63α in chondrocytes and hypertrophic chondrocytes, respectively. These studies support an insignificant role of TAp63α and ΔNp63α in mouse limb development, and suggest the potential importance of other p63 isoforms in skeleton formation [20, 21]. Additional studies strongly indicate an association of TAp63γ with chondrogenesis, as TAp63γ is highly expressed in hypertrophic chondrocytes [13, 22]. However, whether and how TAp63γ plays a role in chondrogenesis during long bone development *in vivo* has not been elucidated.

Here, we used ATDC5 cells, the cell model of endochondral ossification, to perform *in vitro* functional studies involving TAp63γ during chondrogenesis. We have established two stable *TAp63γ* expressing ATDC5 cell lines, pCMV-*TAp63γ* and *Col10a1-TAp63γ*, which are ubiquitously expressed or specifically expressed in hypertrophic chondrocytes. In the early hypertrophic chondrocyte stage, chondrocytes express Col10a1 [26, 27]. We detected significantly upregulated Col10a1 mRNA and protein in both *TAp63γ* stable cell lines compared with the blank and vector controls after 4 and 7 days in culture (Figure 2 and data not shown). Correspondingly, we observed stronger Alcian blue, ALP, and Alizarin red staining in stable cell lines (Figures 3–5), suggesting TAp63γ has a positive effect on Col10a1 expression, chondrocyte proliferation, and hypertrophic differentiation, which are critical components of endochondral ossification.

Both intramembranous ossification and endochondral ossification play a role in vertebrate osteogenesis, and the latter plays a major role in trunk and limb development. In mice at P1, ossification signals in the fore- and hind-limb digits were clearly observed in transgenic mice but were not present or less signals in WT littermates. Moreover, tail ossification signals were evident in caudal vertebra of transgenic mice compared to the controls in two of the transgenic mouse lines (Figure 6). These results indicate that calcified cartilage and ossification zones were enhanced in transgenic mouse limbs. The corresponding increase in the proliferative and hypertrophic zones of the limb cartilage in transgenic mice strongly suggests that TAp63γ promotes chondrocyte hypertrophic differentiation *in vivo* (Figure 7A).

Sox9 plays a critical role in cartilage growth plate development by securing chondrocyte lineage commitment, promoting cell survival, and transcriptionally activating the genes for many cartilage-specific structural components and regulatory factors. Sox9 is essential for permitting chondrocyte proliferation and hypertrophy as well as for inhibiting chondrocyte pre-hypertrophy and ultimate death or osteoblastic transformation [28, 29]. Loss of Sox9 transforms immature chondrocytes into hypertrophic cells [30]. Thus, the decreased Sox9 expression observed in the hypertrophic zone chondrocytes of the long bones in transgenic mice (Figure 7B) may contribute to accelerated chondrocyte differentiation and ossification as well as to promotion of hypertrophy and maturation of chondrocytes. The molecular mechanism of Sox9 regulating chondrogenesis and differentiation has also been reported to be related to the promotion of Runx2 degradation [31]. Moreover, we have detected increased Runx2 expression in the hypertrophic zone in transgenic mice (Figure 7C). Together, the accelerated ossification in our Col10a1–TAp63γ mice could be partially attributed to Runx2 upregulation and Sox9 downregulation which will release the inhibition of Runx2 function to promote chondrocyte maturation and bone formation.

Long bone development is tightly controlled by the activity of the cartilage growth plate. Chondrocyte maturation progresses through the stages of growth plate physiology and eventually reaches chondrocyte hypertrophy, and thus, an essential contributor to longitudinal bone growth. In addition, ectopic hypertrophy of articular chondrocytes has previously been implicated a role in the pathogenesis of osteoarthritis [32]. Our results suggest that TAp63γ plays a positive role in endochondral ossification, possibly through regulation of genes (such as *Alp*, *Sox9*, etc.) relevant to chondrocyte proliferation, maturation, or matrix mineralization. Additional investigation of the role of p63 isoforms during skeletal developmental will increase our understanding of the mechanisms of bone development and the dysfunction that occurs in aging-related joint disease, including osteoarthritis.

## MATERIALS AND METHODS

### Cell lines and cell culture

The ATDC5 cell line was a gift from the department of orthopedic surgery at New York University Medical Center. The ATDC5 cell line is an established *in vitro* model of endochondral ossification that exhibits chondrogenic proliferation, hypertrophic differentiation, and matrix mineralization upon prolonged incubation [4]. ATDC5 cells were cultured in mixed medium containing DMEM/F12 (1:1) with 5% FBS at 37°C and 5% CO_2_ [7, 20].

### Transfection and establishment of stable TAp63γ expressing ATDC5 cell lines

To establish *TAp63γ* expressing stable cell lines, ATDC5 cells were grown at 37°C to 70–80% confluence and were then transfected with a *TAp63γ* expression plasmid or pCMV6-entry as a control. Cells were then cultured with neomycin G418 (600 μg/ml, 158782, MP Biomedicals, Shanghai, China). After 2 weeks, the surviving colonies were picked, and integration of the *TAp63γ* expression plasmid was confirmed. Integrated cells were then used for subsequent experiments.

### Total RNA isolation, RT-PCR, and quantitative real-time-PCR

Total RNA was extracted from proliferative and hypertrophic ATDC5 cells using TRIzol reagent (10296010, Invitrogen, Shanghai, China). Subsequently, cDNA was reverse transcribed from 1 μg of total RNA using Superscript II (Invitrogen) with a total volume of 20 μl, according to the manufacturer’s protocols. Two microliters of diluted (1:10) cDNA sample was used as template for each quantitative real-time PCR (qPCR) reaction to examine expression of following genes: *TAp63γ*, *TAp63γ-Flag*, *Col10a1*, and *Gapdh*. The specific primers for these genes are listed in Table 1. A real-time PCR detection system from Bio-Rad was used to perform qPCR using SYBR Premix Ex Taq II (Bio-Rad, Shanghai, China). One representative data from three independent sets of experiments was selected and analyzed using the comparative 2-^ΔΔ^Ct method [20].

### Western blot

Cells were harvested, homogenized, and lysed in RIPA buffer containing proteinase inhibitors. After centrifugation, protein extracts were quantified by NanoDrop ultraviolet-visible spectrophotometer, and equal amounts of protein (100 μg) were run on SDS-PAGE gels (10%) and transferred onto PVDF membranes. After blocking in 5% nonfat milk in TBS/T for 1 hour, membranes were incubated with primary antibodies, anti-TAp63 (1:500, Biotechnology, Shanghai, China) and anti-Col10a1 (1:150, ab58632, Abcam, UK), at 4°C overnight. The membranes were then incubated with horseradish peroxidase-conjugated secondary antibody (goat anti-rabbit IgG antibody, 1:1000, D110058, Biotechnology, Shanghai, China) for 1 hour and subjected to detection using an enhanced chemiluminescence system (Minichemi, China). Anti-β-actin was used in parallel as the loading control, and experiments were repeated three times.

### Alcian blue, ALP, and Alizarin red staining

ATDC5 cells for Alcian blue staining were fixed with methanol for 2 minutes at -20 °C. After fixation, cells were stained overnight with 0.1% Alcian blue (A0298-1g, Biotechnology, Shanghai, China) in 0.1 N HCL, followed by wash with distilled water. ATDC5 cells were stained for alkaline phosphatase (ALP) according to the instructions provided by the manufacturer (D001-2, Jiancheng, Biotechnology Company Ltd., Nanjing, China). Briefly, cells were washed twice with PBS and fixed with 4% paraformaldehyde for 1 minute, followed by incubation with freshly prepared alkaline phosphatase substrate for 10 minutes at 37°C in a humidified dark box. Cells were washed with PBS and counter-stained with hematoxylin and eosin before microscopic analysis. For Alizarin red staining, cells were washed twice with PBS and fixed with 95% ethanol for 10 minutes prior to staining with 1% Alizarin red (A5333, Sigma, PH 6.4) for 10 minutes at room temperature [33]. Image observation and analysis were collected under Nikon Eclipse 80i microscope (Nikon Instruments Inc., NY, USA). The staining difference was quantified by densitometry analysis of 20 slides using Image-Pro Plus 6 software (Media Cybernetics Inc. USA).

### Generation of Col10a1-TAp63γ transgenic mice

The transgenic construct contained a DDK/Myc flag-tagged *TAp63γ* pCMV plasmid driven by the hypertrophic chondrocyte-specific *Col10a1* regulatory elements that we previously described [21]. The detailed cloning strategy is available upon request. Generation of transgenic mice was conducted at Lannuo Biotechnologies Wuxi, Inc (Wuxi, China). The purified DNA construct was injected into pronuclei of mouse zygotes. The injected zygotes were then transferred into recipient foster mice, and offspring were screened. Transgenic mouse lines were generated as needed [34].

### Genotyping and skeletal phenotypic analysis

PCR genotyping was performed for the *Col10a1-TAp63γ* transgenic mice using DNA prepared from skin. The *p63γ-flag* primer pair (sense, 5′-ACC AGT GAG GTC GTG AGA -3′; antisense, 5′-TCA TTT GCC AGA TCC TCT T-3′) was used for genotyping of *Col10a1-TAp63γ* mice. After genotyping, transgenic and wild-type (WT) littermates at postnatal day 1 (P1) were subjected to skeletal phenotypic analysis. Mouse skeletons were prepared and stained with Alcian blue (8GX, Sigma, St. Louis, MO, USA) and Alizarin red (Sigma, St. Louis, MO, USA) according to published protocols and as previously described [21, 33]. The signal intensity of Alizarin red staining indicates ossification status and was used to evaluate mouse limb, digit, and tail bone ossification (ossified caudal vertebrae numbers).

### Hematoxylin and eosin (H&E) staining

Mouse limbs at P1 were collected, fixed in 10% formalin, and stored in 70% ethanol. Mouse limbs were then subjected to dehydration, paraffin embedding, and sectioning without decalcification. H&E staining was performed using a standard protocol. At least 30 longitudinal (sagittal) sections (5 μm thick) of the limb growth plate from both transgenic and WT littermates were collected and analyzed on a Nikon Eclipse 80i microscope (Nikon Instruments Inc., Melville, NY, USA).

### Immunofluorescent staining

Mouse limb sections at P1 were fixed in 10% formalin, stored in 70% ethanol, and subjected to dehydration, paraffin embedding, and sectioning without decalcification for immunofluorescent analysis. A concentration of 1:100 was used for primary anti-Sox9 (sc-166505, Santa Cruz, CA, USA) and Runx2 (sc-390351, Santa Cruz). Non-immune mouse IgG was used as a negative control. Goat Anti-Mouse IgG H&L (Alexa Fluor® 488) preabsorbed (1:500, ab150117, Abcam) was used as a secondary antibody, and nuclei were stained with 49, 6-diamidino-2-phenylindole (DAPI) for detection. Image observation and analysis were collected under Nikon Eclipse 80i microscope (Nikon Instruments Inc., Melville, NY, USA).

### Statistical analysis

Gene expression was analyzed using GraphPad Prism 5 (GraphPad Software, USA) and student’s *t*-tests. Relative mRNA levels of marker genes were compared using *Gapdh* as an internal control and were quantified using the comparative 2-^ΔΔ^Ct method. P < 0.05 was considered statistically significant.
