# Correction for: TAp63γ influences mouse cartilage development

**DOI:** 10.18632/aging.104221

**Published:** 2020-12-15

**Authors:** Qian Wang, Na Li, Fangzhou Chen, Ruoxuan Hei, Junxia Gu, Yaojuan Lu, Lichun Sun, Qiping Zheng

**Affiliations:** 1 Department of Hematological Laboratory Science, Jiangsu Key Laboratory of Medical Science and Laboratory Medicine, School of Medicine, Jiangsu University, Zhenjiang, 212013, China; 2 Department of Blood Transfusion, The First Affiliated Hospital of Anhui Medical University, Hefei, 230022, China; 3 Shenzhen Academy of Peptide Targeting Technology at Pingshan, and Shenzhen Tyercan Bio-pharm Co., Ltd., Shenzhen, 518118, China; 4 Department of Medicine, School of Medicine, Tulane Health Sciences Center, New Orleans, LA 70112, USA

**Keywords:** Correction

**This article has been corrected:** The authors requested to remove affiliation 1 for Na Li.

The correct affiliation is given below:

Qian Wang^1,*^, Na Li^2,*^, Fangzhou Chen^1^, Ruoxuan Hei^1^, Junxia Gu^1^, Yaojuan Lu^3^, Lichun Sun^4^, Qiping Zheng^1,3^

^1^Department of Hematological Laboratory Science, Jiangsu Key Laboratory of Medical Science and Laboratory Medicine, School of Medicine, Jiangsu University, Zhenjiang 212013, China

^2^Department of Blood Transfusion, The First Affiliated Hospital of Anhui Medical University, Hefei 230022, China

^3^Shenzhen Academy of Peptide Targeting Technology at Pingshan, and Shenzhen Tyercan Bio-pharm Co., Ltd., Shenzhen 518118, China

^4^Department of Medicine, School of Medicine, Tulane Health Sciences Center, New Orleans, LA 70112, USA

*Equal contribution

Original article: Aging. 2020; 12:8669–8679.  . https://doi.org/10.18632/aging.103190

